# Echinacoside Protects Dopaminergic Neurons Through Regulating IL-6/JAK2/STAT3 Pathway in Parkinson’s Disease Model

**DOI:** 10.3389/fphar.2022.848813

**Published:** 2022-02-25

**Authors:** Xueping Yang, Qingyun Yv, Fanlong Ye, Sheng Chen, Zhang He, Wenwei Li, Fang Dong

**Affiliations:** ^1^ Laboratory of Neuropathology and Neuropharmacology, Department of Neurology, Shanghai Public Health Clinical Center, Fudan University, Shanghai, China; ^2^ Institute of Neurology, Institutes of Integrative Medicine, Fudan University, Shanghai, China; ^3^ Department of Breast and Thyroid Surgery, Union Hospital, Tongji Medical College, Huazhong University of Science and Technology, Wuhan, China

**Keywords:** echinacoside, Parkinson’s disease, interleukin-6, JAK2/STAT3, microglia

## Abstract

Echinacoside (ECH), the major active constituent of *Cistanche deserticola*, was found to exert neuroprotection through neurotrophic and anti-inflammatory functions in Parkinson’s disease (PD) models. However, a clear intermediate molecule or pathway that unifies these two effects has to be found. In this study, our results demonstrate that ECH can protect DA neurons in PD mice with Western blot and immunohistochemistry staining. The quantitative real-time polymerase chain reaction was adapted to confirm its anti-inflammatory function with decreased cytokines (interleukin- (IL-) 6, IL-1β, and TNF-α) in PD mice and LPS-induced BV2 cells. Further studies found that ECH inhibited the IL-6/JAK2/STAT3 pathway and decreased phosphorylation of STAT3 on tyr705 by Western blot. It can also increase p-STAT3 (ser727) and brain-derived neurotrophic factor (BDNF) expression in PD mice and LPS-induced BV2 cells. This study revealed that ECH exerts neurotrophic and anti-inflammatory effects by regulating the IL-6/JAK2/STAT3 pathway and the phosphorylation of STAT3, promoting the mutually beneficial influence of the two effects to maximize its neuroprotective function.

## Introduction

Parkinson’s disease (PD) is the second most common neurodegenerative disease, and its most prominent pathological features are the progressive loss of dopaminergic (DA) neurons in substantia nigra (SN) and the Lewy body formed by misfolded α-synuclein (Poewe et al., 2017). While its pathogenesis has not been determined, emerging evidence suggests that microglia-induced chronic neuroinflammation contributes to PD pathogenesis and progression (Ho, 2019).

Previous studies have shown that a microglia activation-triggered release of inflammatory cytokines aggravates the degeneration of DA neurons in the SN (Wang Q et al., 2015). Interleukin-1β (IL-1β) is the most notable pro-inflammatory cytokine involved in neurodegeneration and receives the most attention (Gordon et al., 2018). However, the role of interleukin-6 (IL-6) cannot be underestimated (Spooren et al., 2011). Increased IL-6 levels can be observed in serum of PD patients (Sliter et al., 2018), 1-methyl-4-phenyl-1, 2, 3, 6-tetrahydropyridine- (MPTP-) mediated PD mice (Rojo et al., 2010), 1-methyl-4-phenyl-1,2, 3, 6-tetrahyd-ropyridiniumion- (MPP^+^-) induced SHSY5Y cells (Yao et al., 2019), and BV2 cells (Chen et al., 2015). *In vitro* study, IL-6 causes neuronal cell death (Conroy et al., 2004), and both consecutive low doses (5 × 10 ng/ml) and a single high dose (50 ng/ml) of IL-6 can increase the expression of PD-related protein, α-synuclein (Bick et al., 2008). *In vivo*, the upregulation of IL-6 exacerbates dopaminergic degeneration in 6-hydroxydopamine- (6-OHDA-) induced PD rats (Ma et al., 2020). A four-year prospective study also demonstrated that IL-6, not tumor necrosis factor-α (TNF-α), contributes to mortality in PD Patients (Dufek et al., 2015). Therefore, IL-6 is crucial to neuroinflammation and has emerged as a pivotal player in PD.

During the signal transduction process, IL-6 binds to its receptor and induces the homodimerization of glycoprotein 130 (gp130), mediating the activation of the Janus kinases/signal transducer and activator of transcription proteins (JAKs/STATs) (Garbers et al., 2015). Multiple investigations have shown that IL-6 signaling in the central nervous system (CNS) is orchestrated by STAT-3 (Spooren et al., 2011) with a JAK2-dependent mechanism (Planas et al., 2006). The JAK2/STAT3 activation contributes to cerebral ischemia-perpetuated neuronal damage. However, the intracerebral injection of siRNA specific for STAT3 improves neurological function (Satriotomo et al., 2006). This signal pathway can also be triggered by exposure to manganese in microglia, leading to neuronal loss (Yin et al., 2018). However, microRNA-93 (Wang et al., 2021) and (E)-2-methoxy-4-(3-(4-methoxyphenyl) prop-1-en-1-yl) phenol (Choi et al., 2019) can engender the downregulation of STAT3 to abrogate the MPTP-induced dopaminergic neurodegeneration. Similarly, nitidine has been shown to inhibit the phosphorylation of JAK2/STAT3 and block STAT3 nuclear translocation, exerting neuroprotective effects in PD models (Wang et al., 2016). Therefore the IL-6/JAK2/STAT3 pathway may be the key to PD treatment.

Echinacoside (ECH), the major active constituent of Chinese herb Cistanches Herba (*Cistanche deserticola* Y. C. Ma), reportedly exerts neuroprotective properties in PD models (Chen et al., 2007; Geng et al., 2007; Zhang et al., 2017). There is evidence that ECH also decreases IL-6 expression in the MPTP-induced PD mice (Zhang et al., 2021), ischemia/reperfusion injured rat (Li et al., 2018b), and cervical spondylotic myelopathy rat model (Zhou et al., 2020) and significantly diminishes IL-6 level in 6-OHDA-treated PC12 cells (Wang YH et al., 2015) and lipopolysaccharides- (LPS-) induced BV2 cells (Zhou et al., 2020). Additionally, ECH inhibits the phosphorylation of STAT3 on tyrosine 705 (tyr705) in the LPS-treated rat intestinal epithelial cells (Li et al., 2018a). Both the local knockdown of STAT3 and inhibition of JAK2/STAT3 (tyr705) can restrain acetylated histone 3 (ac-H3) and ac-H4 levels on the promoter of the nucleotide-binding oligomerization domain, leucine-rich repeat, and pyrin domain containing 3 (NLRP3) and decrease the expression of NLRP3 (Zhu et al., 2021), whose activation can also be alleviated by ECH (Zhou et al., 2020). Given these outstanding findings, we hypothesized that ECH’s neuroprotective role is intertwined with the IL-6/JAK2/STAT3 pathway and explored the hypothesis.

## Materials and Methods

### Animals and Drug Administration

The experimental protocols were carried out according to the guidelines for animal experiments of the Shanghai Public Health Center Laboratory Animal Welfare and Ethics Committee (no. 2019-A026-01) to minimize animal suffering. C57BL/6 mice were from the Shanghai Jiesijie Experimental Animal Co., Ltd. (China), housed under a controlled environment (12 h light/dark cycle, 22 ± 2°C, and 55% ± 5% humidity), and provided with food and water *ad libitum.* MPTP·HCL (30 mg/kg, Selleck, United States) were administrated once a day for seven consecutive days to make the subacute mouse model of PD in this study. After two weeks of adaptive feeding, the mice were randomly divided into five groups, namely, normal group (N), MPTP group, MPTP + LECH (LE, 10 mg/kg/d) group, MPTP + MECH (ME, 20 mg/kg/d) group, and MPTP + HECH (HE, 30 mg/kg/d) group. Three doses of ECH treatment were administered the respective pre-treatment ECH (intraperitoneal injection, *i.p.*) every 24 h for 14 consecutive days, with the first day of administration designated as day 1. The mouse PD models for the three treatment groups and the MPTP group were generated by seven consecutive *i.p.* of MPTP (30 mg/kg, Selleck, United States) once a day from day 8 to day 14, as shown below.


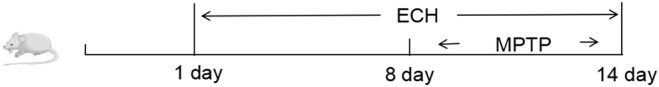


### Open Field Test

The spontaneous locomotor activity of mice was measured with an open field test. Before each behavioral test, the experimental box was scrubbed with 75% ethanol solution to ensure odorless. Then, mice in the different groups were placed into the box, and their behavioral parameters were recorded with a video analyzer for 10 min. The animal behavioral analysis software EthoVisionXT12 (Noldus, the Netherlands) was opened to automatically record the animal movement path, total distance, and other information related to mouse movement.

### Rotarod Test

Mouse motor coordination was evaluated with rotarod apparatus Rotamex-5 Rota Rod (Columbus Instruments, United States). Before the test, all mice were trained on the rotarod (12 rpm) to achieve stable performance. During the test, the mice were placed on the rotarod. Then, it was conducted at a uniformly accelerating speed from 4 to 30 rpm in 300 s. Recording was stopped when the mouse fell off the rotating rod. Repeat the rotarod test three times and take the average time of each mouse to measure its motor ability.

### Pole Test

The pole test was conducted with a rough-surfaced wooden pole (1 cm in diameter, 55 cm in height) to evaluate the bradykinesia of mice. The pole was covered with black tape to prevent the mice from skidding, and a wooden ball (2 cm in diameter) was fixed on the top of the pole. In the beginning, the wooden pole was placed vertically, and a mouse was placed at the top of the pole. Then, it would climb down along the pole to the bottom, and the time used during this period was recorded. The climbing time of mice before ECH treatment (0 days) and after MPTP injection (14 days) was recorded, and the time difference between the two pole tests of the same mouse was calculated.

### Immunohistochemistry Staining

After fixing (4% paraformaldehyde) and dehydration (20% and 30% sucrose solution), brain tissues were sliced into sections with a freezing microtome (Leica Biosystems, Germany) from Bregma −2.00 mm to Bregma −3.52 mm (20 μm for each) and stored in −80°C refrigerator.

After the sections were restored to room temperature, they were treated with 0.3% Triton X-100 to penetrate cytomembrane and 0.3% hydrogen peroxide (H_2_O_2_) to block the endogenous peroxidase activity. Then, they were blocked with bovine serum albumin (BSA) for 30 min and incubated with TH antibody (1:100) overnight at 4°C. On the following day, they were incubated with secondary antibodies (1 h, 37°C) and stained with fresh DAB solution. After placing hematoxylin and hydrochloric acid alcohol, they were dehydrated and dried with gradient alcohol and xylene and sealed with neutral balsam. The images were obtained with the cellSens standard system (Olympus, Japan).

### Immunofluorescence Staining

Immunofluorescence staining was performed with overnight incubation using diluted primary antibodies against IBA-1(1:100, Novus) at 4°C. On the following day, second antibodies were applied to sections and incubated for one hour at room temperature after washing with PBS (three times, 5 min). Then nuclei of cells were counterstained with Hoechst33343 for 2 min.

### Quantitative Real-Time Polymerase Chain Reaction

Total RNA was extracted from SN tissues and BV2 cells with TRIzol reagent according to the standard protocol, respectively, and its purity and concentration were measured with a nucleic acid analyzer (Thermo, United States). Then, it was reverse-transcribed with PrimeScript™ RT reagent Kit with gDNA eraser (Code no. RR047A, Takara, Japan), including genomic DNA remove reaction and reverse transcription reaction. The former system (10 μL) contained 5 × gDNA eraser buffer (2 μL), gDNA eraser (1 μL), total RNA (calculated according to concentration), and RNase-free dH_2_O (up to 10 μL), and the latter (total 20 μL) included the reaction liquid from last step (10 μL), PrimeScript RT Enzyme Mix I (1 μL), RT Primer Mix *4 (1 μL), 5 × PrimeScript Buffer 2 (4 μL), and RNase-free dH_2_O (4 μL). The RT-qPCR system was configured with TB Green^®^ Premix Ex Taq™ (Tli RNaseH Plus) (Code no. RR420A, Takara, Japan) on ice, and the RT-qPCR was run with CFX96 Real-Time PCR Detection System (Bio-Rad, United States). The mRNA levels of target genes were normalized to that of β-actin in the same sample. The primer sequences in RT-qPCR are shown in Table 1.

**TABLE 1:** Real-time PCR primer sequences.

Gene	Forward (5′-3′), reverse (3′-5′)
IL-1β	CAC​TAC​AGG​CTC​CGA​GAT​GAA​C TCC​ATC​TTC​TTC​TTT​GGG​TAT​TGC
TNF-α	ACC​CTC​ACA​CTC​ACA​AAC​CA ATA​GCA​AAT​CGG​CTG​ACG​GT
IL-6	TTC​TTG​GGA​CTG​ATG​CTG​GTG CAC​AAC​TCT​TTT​CTC​ATT​TCC​ACG​A
β-Actin	GTG​ACG​TTG​ACA​TCC​GTA​AAG​A GTA​ACA​GTC​CGC​CTA​GAA​GCA​C

### Cell Culture

BV2 cells, provided by the State Key Laboratory of Medical Neurobiology, Fudan University, were maintained in high-glucose Dulbecco’s modified Eagle’s medium (DMEM, HyClone) with 10% fetal bovine serum (FBS, HyClone, United States), 100 U/mL penicillin, and 100 g/ml streptomycin in a 5% CO_2_ incubator (37°C).

### Chemicals and Reagents

Echinacoside was purchased from Selleck and dissolved into 5, 10, and 20 mg/L. LPS were from Sigma. STAT3 inhibitor, S3I-201, was purchased from Selleck. Opti-MEM was from Gibco. Antibodies used in this study such as those against tyrosine hydroxylase (TH), against α-synuclein, and against brain-derived neurotrophic factor (BDNF) were purchased from Cell Signaling Technology (CST, United States); that against ionized calcium-binding adapter molecule 1 (IBA-1, goat) was from Novus Bio; those against STAT3, p-STAT3(tyr705), p-STAT3(ser727), donkey anti-rabbit IgG (Alexa Fluor^®^ 594), and donkey anti-goat IgG (Alexa Fluor^®^ 488) were from Abcam (United States); and those against IL-6, p-JAK2 (tyr1007/1008), JAK2, and β-actin were from ABclonal (China). SDS-PAGE gel preparation and cocktail protease inhibitor were purchased from Servicebio (China). Universal antibody diluent and serum-free cell freezing medium were purchased from New Cell and Molecular Biotech (NCM, China). Protein Ladder and SuperSignal West Pico PLUS Chemiluminescent Substrate were purchased from Thermo (United States).

### Cell Viability

Cell survival was assayed with cell counting kit-8 (CCK8) according to the manufacturer’s instructions (Beyotime). BV2 microglial cells were plated into 96-well plates. After planning LPS and ECH intervention, CCK-8 solution was added into each well, followed by incubation at 37°C for 2 h. The absorbance of different wells was measured at 450 nm with a Microplate photometer (Thermo, United States).

### Western Blot Analysis

The proteins of SN tissues and BV2 cells were extracted with lysates supplemented with protease inhibitor cocktail and phosphatase inhibitor, and the protein expression was measured with Western blot. Then, the concentrated gel and separated gel were prepared according to the instructions. The proteins were separated on 10%–12% SDS-PAGE (80V, 0.5 h; then 120 V, 1 h) and transferred to 0.22 μm PVDF membrane. Membranes were blocked with BSA (5%, 1 h, room temperature) and then incubated with primary antibodies (4°C, overnight). On the following day, secondary antibodies (1 h, room temperature) were applied to the membrane, followed by three-time washes with Tris-buffered saline and Tween 20 (TBST, 10 min each). Bands were visualized with ChemiScope 6000 Exp (Clinx Science Instruments, China) and normalized against β-actin with Image-Pro Plus software (Media Cybernetics, United States).

### Statistical Analysis

Statistical analyses of data were performed with the SPSS 19.0 software program (Chicago, IL, United States). The significance of differences between groups was evaluated with the one-way analysis of variance (ANOVA) and Dunnett’s *t*-test, and *p* < 0.05 was considered significant.

## Result

### Echinacoside Protected Mice Against MPTP-Induced Behavioral Dysfunction

**FIGURE 1:**
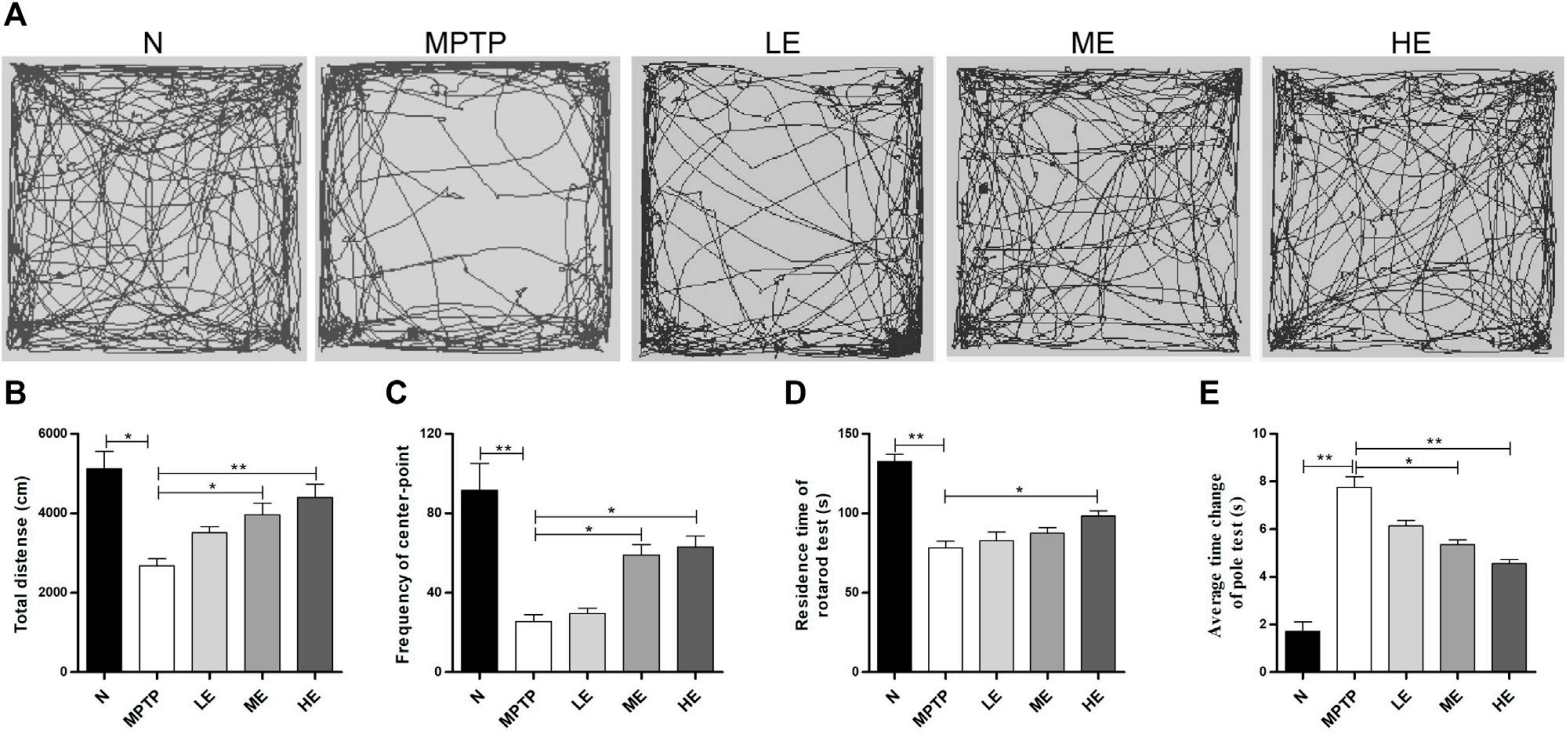
Protective effect of ECH against MPTP-induced behavioral dysfunction. **(A)** The autonomous trajectory map of mice in the open field test. **(B)** Total distance traveled of mice in the open field test. **(C)** The number of line crossings of mice in the open field test **(D)** The residence time of mice in the rotarod test. **(E)** The average time change of the pole test. N: normal; MPTP: MPTP-induced mice; LE: MPTP + 10 mg/kg/d ECH; ME: MPTP + 20 mg/kg/d ECH; HE: MPTP + 30 mg/kg/d ECH. Values are presented as the mean ± SEM. ^*^ *p* < 0.05, ^**^ *p* < 0.01.

We assessed the establishment of neurobehavior in mice with the open field, rotarod, and pole tests. For the open field test, we recorded the autonomous activity tracks of mice with EthoVision XT12, as shown in Figure 1A. Compared with the control group, the motor complexity in the MPTP group was significantly reduced, resulting in decreases in the total distance (Figure 1B) and the number of line crossings (Figure 1C). However, treatment with ECH reversed all three actions.

We evaluated the exercise coordination ability of mice with the rotarod test. As shown in Figure 1D, the residence time in the model group was shorter than that in the control group. Of the three different treatment doses, only the high ECH doses prolonged the duration of MPTP mice on the rotarod.

Per the pole test findings, the descending time from the top to the bottom of the pole was markedly lengthier in the MPTP group than in the control group. However, this phenomenon was remedied by intervention with ECH (Figure 1E).

### Echinacoside Protected Dopaminergic Neurons and Decreased the Expression of α-Synuclein in MPTP-Induced Parkinson’s Disease Mice

**FIGURE 2:**
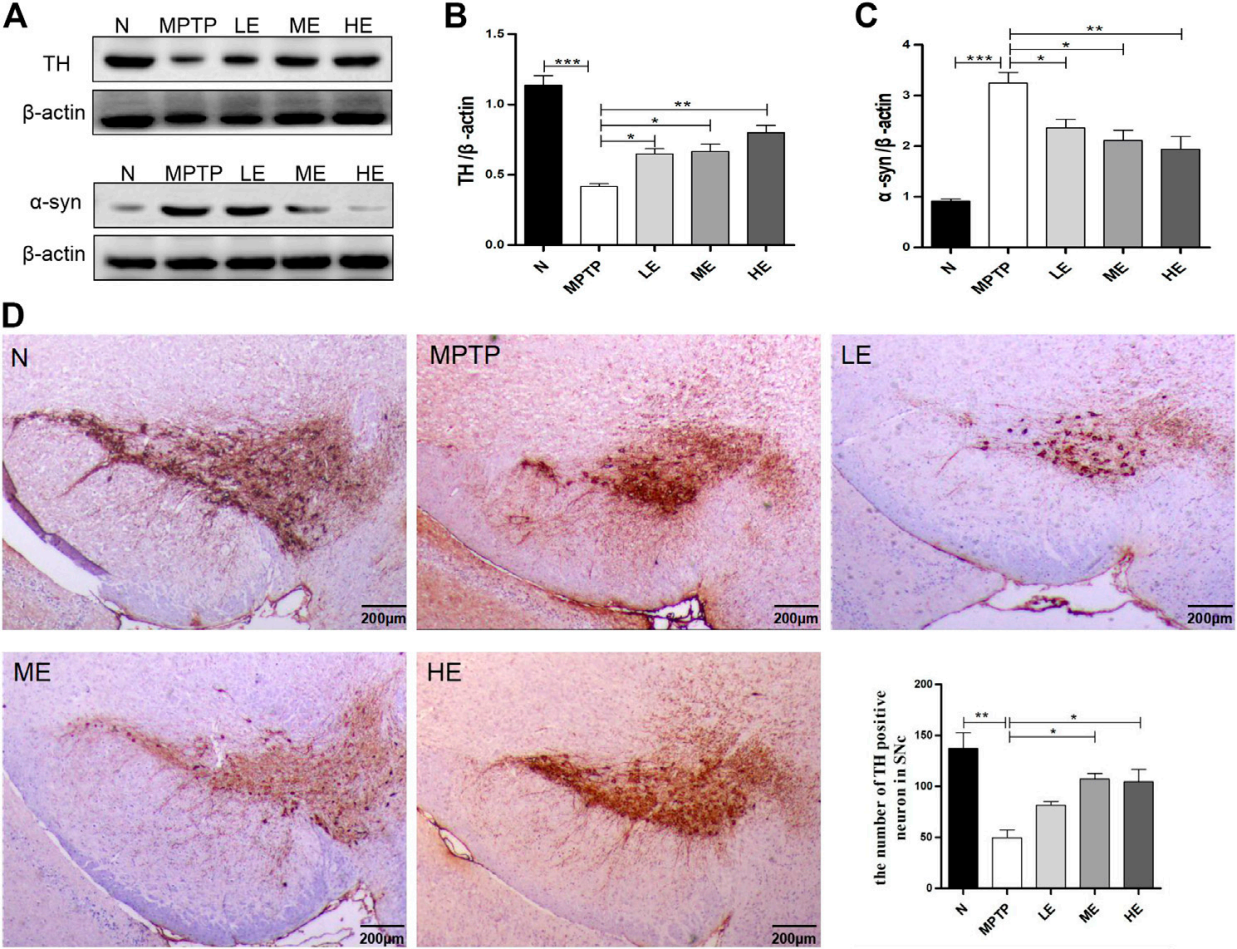
ECH protected DA neurons and decreased the expression of α-synuclein in substantia nigra (SN) of MPTP-induced mice. **(A)** Expression of TH and α-synuclein of mice in different groups. **(B)** Data analysis of TH expression. **(C)** Data analysis of α-synuclein expression. **(D)** Representative microphotographs of dopaminergic neurons stained for TH. Scale bars: 200 μm. Values are presented as the mean ± SEM. ^*^ *p* < 0.05, ^**^ *p* < 0.01, ^***^ *p* < 0.001.

To assess the protective effect of ECH on the nigrostriatal DA system, we examined the expression of TH and α-synuclein in the SN. Mice in the MPTP-induced group showed reduced TH expression (Figures 2A,B) and increased α-synuclein deposition (Figure 2A,C) compared to those in the control group; however, these trends were reversed with ECH treatment. Consistent with Western blot findings, immune-histochemistry also demonstrated that mice in the MPTP group exhibited reduced TH-positive neurons in the SN by approximately 64% compared to mice in the control group, but treatment with ECH reversed this condition (Figure 2D).

### Echinacoside Inhibited the Activation of Microglia and Secretion of Inflammatory Cytokines in MPTP-Induced Parkinson’s Disease Mice

**FIGURE 3:**
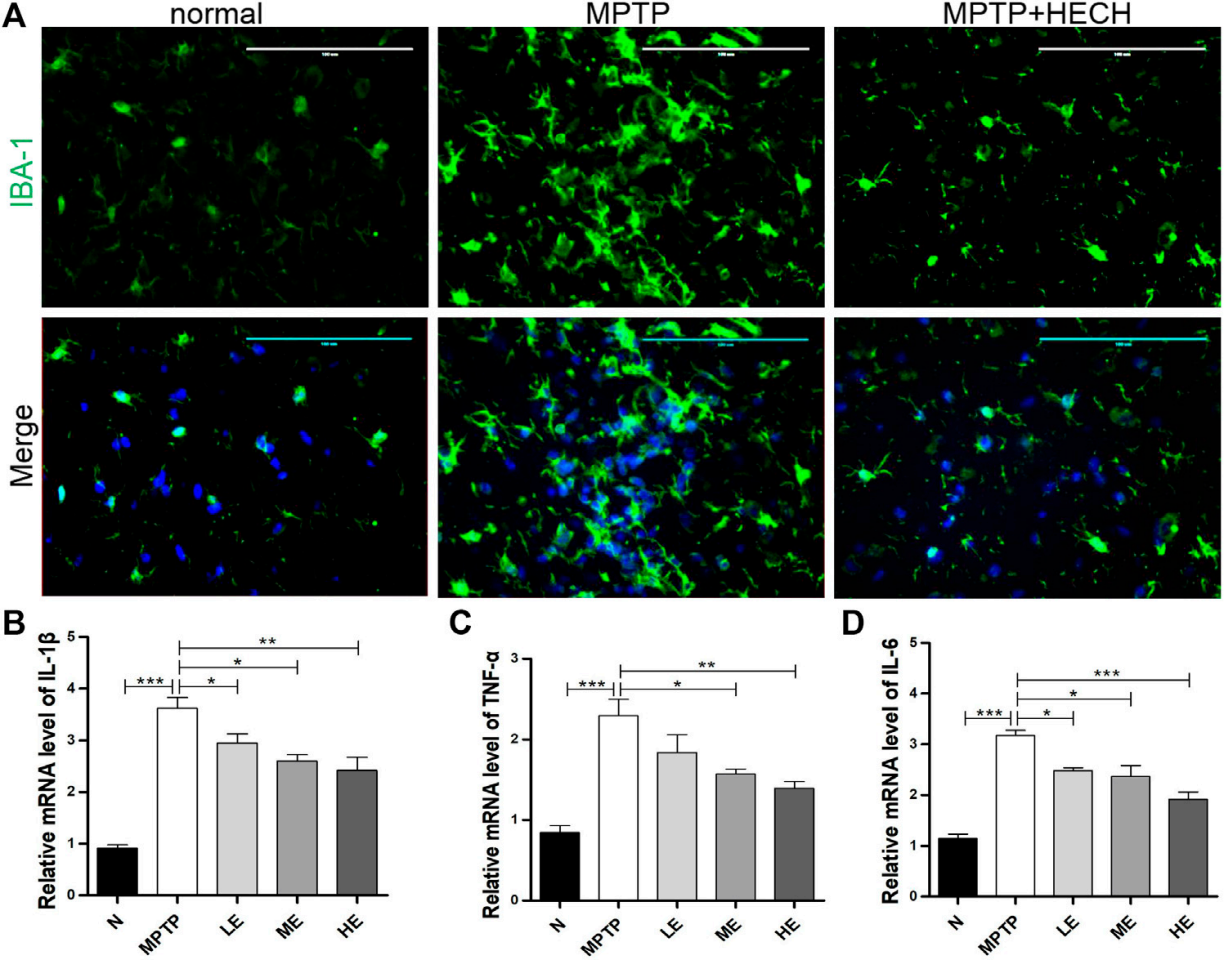
ECH inhibited the activation of microglia and secretion of inflammatory cytokines in SN of MPTP mice. **(A)** Representative photos of immunofluorescence staining of IBA-1 in the striatum. **(B)** Relative mRNA level of IL-1β in SN of different groups. **(C)** Relative mRNA level of TNF-α in SN of different groups. **(D)** Relative mRNA level of IL-6 in SN of different groups. Values are presented as the mean ± SEM. ^*^ *p* < 0.05, ^**^ *p* < 0.01, ^***^ *p* < 0.001.

Then, we performed the immunofluorescence staining of IBA-1 and observed significantly positive microglial staining in the SN of MPTP mice more than in the same areas of control mice (Figure 3A) indicating that microglia were activated in the MPTP group mice; however, ECH inhibited the activation of microglia.

Because pro-inflammatory cytokines play a crucial role in neuroinflammation-related PD, we conducted RT-qPCR on SN homogenates and found IL-1β (Figure 3B), TNF-α (Figure 3C), and IL-6 (Figure 3D) expressions markedly increased in the MPTP-induced mice compared to control mice. In contrast, treatment with medium and high ECH doses reduced the production of these cytokines to different degrees.

### Echinacoside Regulated IL-6/JAK2/STAT3 Signaling in MPTP-Induced Parkinson’s Disease Mice

**FIGURE 4:**
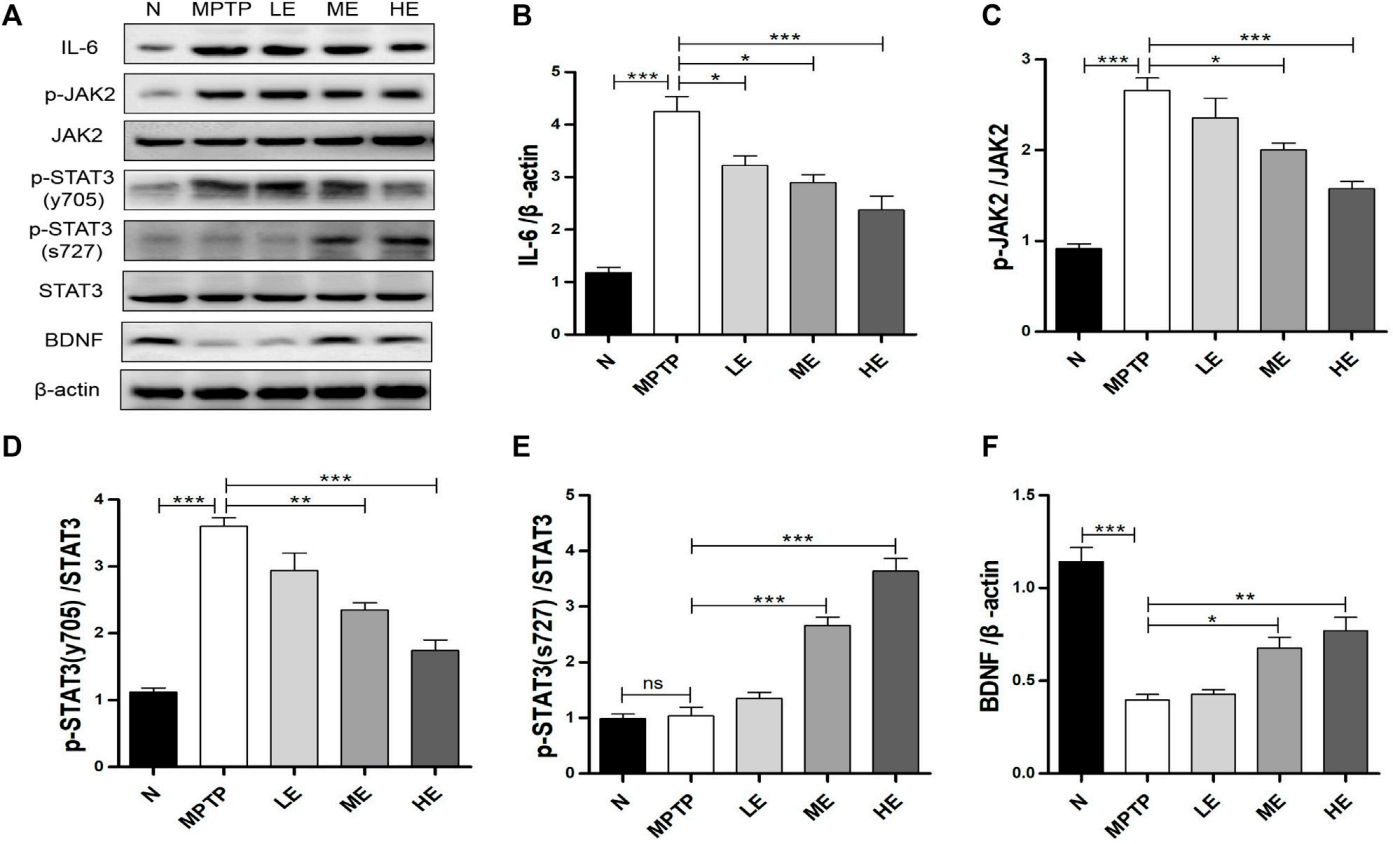
ECH regulated IL-6/JAK2/STAT3 signaling in SN of MPTP mice. **(A)** Expression of IL-6, p-JAK2, JAK2, p-STAT3 (tyr705), p-STAT3 (ser727), STAT3, and BDNF in SN. **(B)** Western blot analysis for IL-6. **(C)** Western blot analysis for p-JAK2/JAK. **(D)** Western blot analysis for p-STAT3 (tyr705)/STAT3. **(E)** Western blot analysis for p-STAT3 (ser727)/STAT3. **(F)** Western blot analysis for BDNF. Values are presented as the mean ± SEM. ^*^ *p* < 0.05, ^**^ *p* < 0.01, ^***^ *p* < 0.001.

As the downstream of IL-6, we analyzed the activation of JAK2/STAT3 signaling and the phosphorylation of STAT3 (tyr705) with Western blot. As shown in Figure 4A, MPTP triggered considerable augmentations in IL-6, p-JAK2, and p-STAT3 (tyr705) expressions in the SN; however, ECH suppressed these elevations dose-dependently (Figures 4A–D). Notably, MPTP induced phosphorylation at the tyr705 site of STAT3, not the ser727 site, as the latter was not detected, as shown in Figure 4A, which is consistent with findings from a previous study (Sriram et al., 2004). In contrast, ECH activated STAT phosphorylation on ser727 (Figure 4E) and upregulated BNDF expression in the SN of mice (Figure 4F).

### Echinacoside Retrained the Activation of Microglia and Secretion of Inflammatory Cytokines in Lipopolysaccharide-Induced BV2 Cells

**FIGURE 5:**
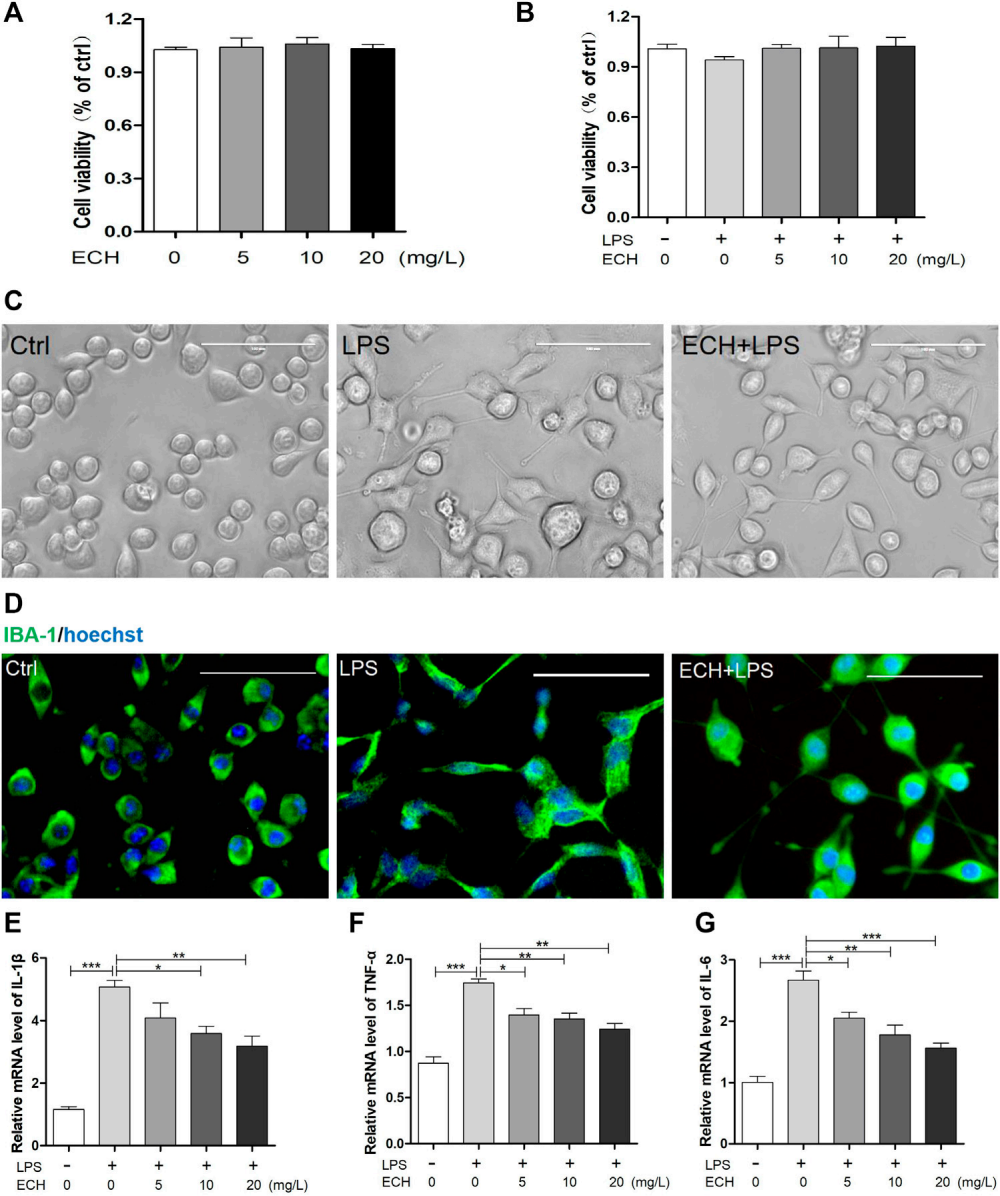
ECH retrained the activation of microglia and secretion of inflammatory cytokines in LPS-induced BV2 cells. **(A)** BV2 microglial cells were treated with ECH (5, 10, and 20 mg/L) for 2 h, and cell viability was measured by CCK8. **(B)** BV2 microglial cells were pretreated with ECH (5, 10, and 20 mg/L) for 2 h and exposed to LPS (1 μg/ml) for 6 h, and cell viability was measured. **(C)** Morphology of BV2 cells in different groups. **(D)** Representative photos of immunofluorescence staining of IBA-1 in BV2 cells. **(E)** The relative mRNA level of IL-1β in different groups. **(F)** Relative mRNA level of TNF-α. **(G)** Relative mRNA level of IL-6. Values are presented as the mean ± SEM. ^*^ *p* < 0.05, ^**^ *p* < 0.01, ^***^ *p* < 0.001.

We investigated the impact of ECH on the activation of LPS-treated (1 μg/ml) BV2 microglial cells *in vitro*. To assess the effect of ECH on cell viability, we added ECH (5, 10, and 20 mg/L) into cell culture media alone or alongside LPS. As shown in Figures 5A,B, treatment with ECH and LPS did not alter cell viability compared to vehicle. Morphological changes were observed in BV2 cells under a light microscope (Figure 5C). BV2 cells in the LPS group were larger, harbored shorter processes, and displayed apparent swelling of the cell bodies compared to the control group. However, treatment with ECH significantly reversed these morphological changes. Figure 5D, which depicts the activation of BV2 cells *via* the immunofluorescence staining of IBA-1, exhibits a similar trend.

To determine ECH’s ability to affect LPS-induced neuroinflammation, we treated BV2 microglial cells with ECH (5, 10, and 20 mg/L) for 2 h, followed by LPS (1 μg/ml) for 6 h, and then determined their pro-inflammatory cytokine levels with RT-PCR. As shown in Figures 5E–G, ECH markedly decreased the mRNA level of LPS-induced pro-inflammatory cytokines IL-1β, TNF-α, and IL-6.

### Echinacoside Regulated IL-6/JAK2/STAT3 Signaling in Lipopolysaccharide-Induced BV2 Cells

**FIGURE 6:**
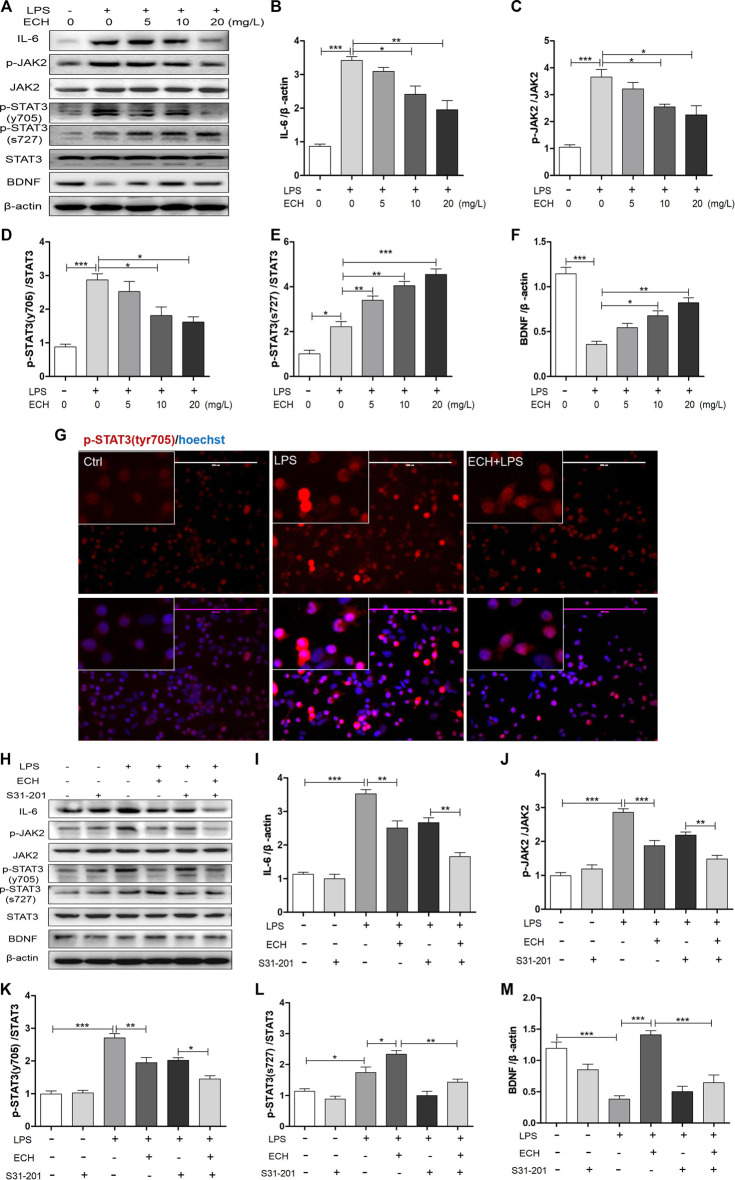
ECH regulated IL-6/JAK2/STAT3 signaling in LPS-induced BV2 cells. **(A)** BV2 microglial cells were pretreated with ECH (5, 10, and 20 mg/L) for 2 h and exposed to LPS (1 μg/ml) for 6h; then, the expression levels of IL-6, p-JAK2, JAK2, p-STAT3 (tyr705), p-STAT3 (ser727), STAT3, and BDNF were measured with Western blot. **(B)** Western blot analysis for IL-6. **(C)** Western blot analysis for p-JAK2/JAK. **(D)** Western blot analysis for p-STAT3/STAT3. **(E)** Western blot analysis for p-STAT3 (ser727)/STAT3. **(F)** Western blot analysis for BDNF. **(G)** Immunofluorescence staining for p-STAT3 (tyr705) (red) in ECH (20 mg/L, 2 h) pretreated BV2 cells and then exposed to LPS (1 μg/ml) for 6 h. **(H)** BV2 cells were pretreated with S3I-201 (STAT3 inhibitor, 100 µM) for 24 h and then treated with ECH (20 mg/L) for 2 h and induced by LPS (1 μg/ml) for 6 h. The expression levels of IL-6, p-JAK2, JAK2, p-STAT3 (tyr705), p-STAT3 (ser727), STAT3, and BDNF are shown under S3I-201 treatment. **(I)** Western blot analysis for IL-6. **(J)** Western blot analysis for p-JAK2/JAK. **(K)** Western blot analysis for p-STAT3 (tyr705)/STAT3. **(L)** Western blot analysis for p-STAT3 (ser727)/STAT3. **(M)** Western blot analysis for BDNF. Values are presented as the mean ± SEM. ^*^ *p* < 0.05, ^**^ *p* < 0.01, ^***^ *p* < 0.001.

We examined the impact of ECH on IL-6/JAK2/STAT3 signaling in LPS-induced BV2 cells. As shown in Figure 6A, LPS administration led to the enhanced expression of IL-6, p-JAK2, and p-STAT3 (tyr705) proteins. Co-treatment with different doses of ECH saw a decline in the increased expressions of these three proteins dose-dependently; their Western blot analyses are shown in Figures 6B–D, respectively. Unlike in MPTP mice, p-STAT3 (ser727) was also activated in LPS-treated BV2 cells, followed by a persistent and dose-dependent upregulation after treatment with different ECH doses (Figure 6E). As a result, ECH treatments also amplified BDNF expression in the same trend as in MPTP mice (Figure 6F).

We conducted cellular immunofluorescence assays of p-STAT3 (tyr705) to observe the nuclear transcription of STAT3 directly. As shown in Figure 6G, compared with the control group, the STAT3 (tyr705) level in nuclei was significantly upregulated in LPS-induced BV2 cells; however, ECH significantly reduced these levels.

We used the inhibitor of STAT3, S3I-201, to examine the ECH’s regulation of IL-6/JAK2/STAT3 (tyr705) and BNDF/STAT3 (ser727). BV2 cells were pretreated with S3I-201 (100 μM) for 24 h and then treated with ECH (20 mg/L) for 2h, followed by LPS (1 μg/ml) for 6 h. IL-6, p-JAK2, and p-STAT3 expressions were determined with Western blot (Figure 6H). Treatment with “S3I-201 + ECH + LPS” further decreased LPS-stimulated IL-6, p-JAK2, and p-STAT3 (tyr705) protein expressions compared to treatment with “S3I-201 + LPS” (Figures 6I–K). Additionally, treatment with S3I-201 eliminated ECH-engendered p-STAT3 (ser727) (Figure 6L) and BDNF upregulation (Figure 6M).

## Discussion

Due to the spiraling prevalence of PD, progressively more scholars are paying attention to the impact of natural drugs and their extracts on PD (Rabiei et al., 2019). Epidemiological study findings suggest that some patients with PD do not have tremors, even if they all develop bradykinesia. The movement disorder society’s (MDS) clinical diagnostic criteria for PD also clearly stated in its report that bradykinesia was the only critical symptom of PD in 2015 (Postuma et al., 2015), suggesting that the “kidney-qi deficiency” (a traditional Chinese medicine diagnostic statement) associated with bradykinesia could be pivotal to the pathogenesis of PD. Therefore, *Cistanche deserticola* with the “kidney tonifying” effect has been widely studied in the search for drugs against PD (Gu et al., 2016), with evidence that its anti-PD activity comes from its phenylethanoid glycoside extract (Fu et al., 2018). ECH, a phenylethanoid glycoside derived from *Cistanche deserticola*, exerts neuroprotective properties through neurotrophic (Zhu et al., 2013) and anti-inflammatory (Zhang et al., 2021) functions. However, a clear intermediate molecule or pathway that unifies these two effects has to be found. We reveal here that ECH can achieve neurotrophic and anti-inflammatory effects by regulating the IL-6/JAK2/STAT3 pathway and the phosphorylation of STAT3, promoting the mutually beneficial influence of the two effects to maximize its neuroprotective function.

IL-6 was uncovered about forty years ago, initially as an inflammatory cytokine produced by lymphocytes involved in B-cell differentiation (Hirano et al., 1986). Its persistent synthetic dysregulation pathologically affects autoimmunity and chronic inflammation (Tanaka et al., 2014). Alongside TNF-α and IL-1β, IL-6 is considered one of the chief orchestrators of the inflammatory responses in the brain and has emerged as a pivotal player in the nervous system (Qin et al., 2016). Its production can be stimulated by neurotransmitters, α-synuclein, inflammatory cytokines (e.g., TNF-α, IL-1β, and IL-6), and bacterial pathogens (e.g., LPS) (Spooren et al., 2011). Reports show that LPS-induced microgliosis is blocked by the intracerebral injection of an anti-IL-6 antibody (Pang et al., 2006), and microglial activation is reduced in IL-6^−/−^ mice (Galiano et al., 2001). In this study, the IL-6 level was decreased with ECH treatment in LPS-induced BV2 cells. Allegedly, IL-6 is also a neurotrophic factor thanks to its impact on the survival, proliferation, and differentiation of neurons (Satoh et al., 1988). IL-6 at low concentrations can induce neuronal survival and outgrowth in LPS treated-microglia (Li et al., 2007) or astrocytes- (Li et al., 2009) conditioned media, and at high concentrations, it can cause neuronal death; however, these phenomena can be arrested with an IL-6 antibody, suggesting that the neuroprotective effect of IL-6 is conditional and dependent on the environment and concentration. Excessive IL-6 can also enhance N-methyl-D-aspartic (NMDA) acid receptor mediated neuronal excitotoxicity (Qiu et al., 1998), which can equally be induced by MPTP (Wang et al., 2010), ultimately leading to neurodegeneration. The PD-associated protein α-synuclein is likewise upregulated in the presence of high dose IL-6 (Bick et al., 2008). Therefore, excessive dysregulations of IL-6 in MPTP-induced PD mice and LPS-treated BV2 cells can produce deleterious effects controllable with ECH to protect DA neurons, as shown in this study.

During signal transduction, IL-6 binds to its receptor, which induces and induces the homodimerization of gp130 followed by activation of the associated JAKs (Garbers et al., 2015). Subsequently, JAKs phosphorylate STATs and then form homologous or heterodimers that translocate to the nucleus, bind to specific DNA sequences, and promote transcriptional activation (Planas et al., 2006). Both STAT1 and STAT3 can be activated by IL-6 *in vitro* (Jenab and Quinones-Jenab, 2002), while *in vivo* studies revealed that STAT1 has a limited role in IL-6 signaling (Sanz et al., 2008). Studies have demonstrated that IL-6 signaling in the CNS is coordinated by STAT-3 (Spooren et al., 2011), with a JAK2-dependent mechanism (Planas et al., 2006).

As the downstream effector of IL-6, the JAK2/STAT3 pathway can be activated in response to the reactive oxygen species (ROS) and pro-inflammatory cytokines, TNF-α and IL-1β (Dziennis and Alkayed., 2008). STAT3 activity depends primarily on its phosphorylation at tyr705, which is regulated by the activities of the tyrosine kinases and tyrosine phosphatases of STAT3 (Mertens and Darnell., 2007), and then it was transported to the nucleus to trigger gene transcription. Activating JAK2/STAT3 pathway initiates the activation of microglia, resulting in the neurodegeneration of dopaminergic neurons (Huang et al., 2008). It can also be induced by the overexpression of α-synuclein, ultimately contributing to neurodegeneration. JAK2/STAT3 signaling inhibition has been shown to protect against α-synuclein-induced neuroinflammation and dopaminergic neurodegeneration in the α-synuclein overexpression PD model (Qin et al., 2016). Per previous reports, JAK2 is activated and STAT3 is phosphorylated on tyr705 in MPTP-induced PD mice (Sriram et al., 2004) and LPS-treated BV2 microglial cells (Li et al., 2016), consistent with related results in the current study.

In addition to the JAK2-induced phosphorylation on tyr705, the phosphorylation of STAT3 on ser727 also appears to participate in the regulation of STAT3 activation. Reportedly, neurotrophins and cytokines stimulate STAT3 phosphorylation on ser727 and tyr705, respectively, in sympathetic neurons (Pellegrino and Habecker, 2013). The nerve growth factor (NGF) can activate STAT3 through TrkA to target the phosphorylation of STAT3 on ser727 but not on tyr705 (Pellegrino and Habecker, 2013; Ng et al., 2006). Similarly, the BDNF-engendered activation of TrkB also leads to STAT3 phosphorylation on ser727 (Zhou & Too, 2011). Single-cell transcriptome analyses identified BDNF as a STAT3 target gene, with its expression capable of increasing with the activation of p-STAT3 (Paris et al., 2020), pointing to a mutually promoting relationship between BDNF and p-STAT3 (ser727). Reportedly, transient amounts of ECH can heighten TrkA/TrkB activity and increase NGF/BDNF in rotenone-treated primary rat cortical neurons (Zhu et al., 2013), and ECH initiates a rise in BDNF in the SN of MPTP-induced PD mice (Zhang et al., 2021). In this study, treatment with ECH increased BDNF expression in MPTP mice and LPS-treated BV2 cells, suggesting that ECH possibly also activates STAT3 phosphorylation on ser727, as shown in Figures 4E, 6E. According to past investigations, p-STAT3 (ser727) enhancement is associated with p-STAT3 (tyr705) downregulation (Shi et al., 2006; Andersson et al., 2007). This negative relationship between p-STAT3 (ser727) and p-STAT3 (tyr705) has been observed before, where casein kinase 2-mediated reduction in STAT3 phosphorylation at the Ser727 site triggered an increase in STAT3 phosphorylation on Tyr705 (Mandal et al., 2014). In this study, ECH caused p-STAT3 (tyr705) downregulation in MPTP mice and LPS-treated BV2 cells, as depicted in Figures 4, 6. ECH, therefore, possibly plays dual neurotrophic and anti-inflammatory roles by upregulating p-STAT3 (Ser727) and downregulating p-STAT3 (tyr705), respectively, protecting damaged neurons in PD mice models.

## Data Availability

The original contributions presented in the study are included in the article/Supplementary Materials, further inquiries can be directed to the corresponding authors.

## Abbreviations

6-OHDA, 6-hydroxydopamine; ac-H3, acetylated histone 3; BDNF, brain-derived neurotrophic factor; BSA, bovine serum albumin; CCK8, cell counting kit-8; CNS, central nervous system; DA, dopaminergic; DOPAC, dihydroxyphenylacetic acid; DMEM, Dulbecco’s modified Eagle’s medium; ECH, echinacoside; ELISA, enzyme-linked immunosorbent assay; FBS, fetal bovine serum; IBA-1, ionized calcium-binding adapter molecule 1; IL-1β, interleukin-1β; IL-6, interleukin-6; ip, intraperitoneal injection; JAK, Janus kinases; LPS, lipopolysaccharides; MDS, movement disorder society; MPTP, 1-methyl-4-phenyl-1,2,3,6-tetrahydropyridine; NLRP3, nucleotide-binding oligomerization domain, leucine-rich repeat and pyrin domain containing 3; NGF, nerve growth factor; NMDA, N-methyl-D-aspartic acid receptor; PBS, phosphate-buffered saline; PD, Parkinson’s disease; ROS, reactive oxygen species; RT-qPCR, quantitative real-time polymerase chain reaction; Ser727, Serine 727; SN, substantia nigra; STAT, signal transducer and activator of transcription protein; SIRT1, sirtuin-1; TBST, Tris-buffered saline and Tween 20; TH, tyrosine hydroxylase; TNF-α, tumor necrosis factor-α; tyr705, tyrosine 705.

## References

[B1] Andersson C. X. Sopasakis V. R. Wallerstedt E. Smith U. (2007). Insulin Antagonizes Interleukin-6 Signaling and Is Anti-inflammatory in 3T3-L1 Adipocytes. J. Biol. Chem. 282 (13), 9430–9435. 10.1074/jbc.M609980200 17267401

[B2] Bick R. J. Poindexter B. J. Kott M. M. Liang Y. A. Dinh K. Kaur B. (2008). Cytokines Disrupt Intracellular Patterns of Parkinson's Disease-Associated Proteins Alpha-Synuclein, Tau and Ubiquitin in Cultured Glial Cells. Brain Res. 1217, 203–212. 10.1016/j.brainres.2008.03.081 18501880

[B3] Chen H. Jing F. C. Li C. L. Tu P. F. Zheng Q. S. Wang Z. H. (2007). Echinacoside Prevents the Striatal Extracellular Levels of Monoamine Neurotransmitters from Diminution in 6-hydroxydopamine Lesion Rats. J. Ethnopharmacol 114, 285–289. 10.1016/j.jep.2007.07.035 17951018

[B4] Chen T. Hou R. Xu S. Wu C. (2015). Donepezil Regulates 1-Methyl-4-Phenylpyridinium-Induced Microglial Polarization in Parkinson's Disease. ACS Chem. Neurosci. 6 (10), 1708–1714. 10.1021/acschemneuro.5b00026 26114860

[B5] Choi J. Y. Yun J. Hwang C. J. Lee H. P. Kim H. D. Chun H. Park P. H. Choi D. Y. Han S. B. Hong J. T. (2019). (E)-2-methoxy-4-(3-(4-methoxyphenyl) Prop-1-En-1-Yl) Phenol Ameliorates MPTP-Induced Dopaminergic Neurodegeneration by Inhibiting the STAT3 Pathway. Int. J. Mol. Sci. 20 (11), 2632. 10.3390/ijms20112632 PMC6600543 31146332

[B6] Conroy S. M. Nguyen V. Quina L. A. Blakely-Gonzales P. Ur C. Netzeband J. G. (2004). Interleukin-6 Produces Neuronal Loss in Developing Cerebellar Granule Neuron Cultures. J. Neuroimmunol 155, 43–54. 10.1016/j.jneuroim.2004.06.014 15342195

[B7] Dufek M. Rektorova I. Thon V. Lokaj J. Rektor I. (2015). Interleukin-6 May Contribute to Mortality in Parkinson's Disease Patients: A 4-Year Prospective Study. Parkinsons Dis. 2015, 898192. 10.1155/2015/898192 26351617 PMC4553204

[B8] Dziennis S. Alkayed N. J. (2008). Role of Signal Transducer and Activator of Transcription 3 in Neuronal Survival and Regeneration. Rev. Neurosci. 19, 341–361. 10.1515/revneuro.2008.19.4-5.341 19145989 PMC2681485

[B9] Fu Z. Fan X. Wang X. Gao X. (2018). Cistanches Herba: An Overview of its Chemistry, Pharmacology, and Pharmacokinetics Property. J. Ethnopharmacol 219, 233–247. 10.1016/j.jep.2017.10.015 29054705

[B10] Galiano M. Liu Z. Q. Kalla R. Bohatschek M. Koppius A. Gschwendtner A. (2001). Interleukin-6 (IL6) and Cellular Response to Facial Nerve Injury: Effects on Lymphocyte Recruitment, Early Microglial Activation and Axonal Outgrowth in IL6-deficient Mice. Eur. J. Neurosci. 14 (2), 327–341. 10.1046/j.0953-816x.2001.01647.x 11553283

[B11] Garbers C. Aparicio-Siegmund S. Rose-John S. (2015). The IL-6/gp130/STAT3 Signaling axis: Recent Advances towards Specific Inhibition. Curr. Opin. Immunol. 34, 75–82. 10.1016/j.coi.2015.02.008 25749511

[B12] Geng X. Tian X. Tu P. Pu X. (2007). Neuroprotective Effects of Echinacoside in the Mouse MPTP Model of Parkinson's Disease. Eur. J. Pharmacol. 564, 66–74. 10.1016/j.ejphar.2007.01.084 17359968

[B13] Gordon R. Albornoz E. A. Christie D. C. Langley M. R. Kumar V. Mantovani S. (2018). Inflammasome Inhibition Prevents α-synuclein Pathology and Dopaminergic Neurodegeneration in Mice. Sci. Transl Med. 10, eaah4066. 10.1126/scitranslmed.aah4066 30381407 PMC6483075

[B14] Gu C. Yang X. Huang L. (2016). Cistanches Herba: A Neuropharmacology Review. Front. Pharmacol. 7, 289. 10.3389/fphar.2016.00289 27703431 PMC5028387

[B15] Hirano T. Yasukawa K. Harada H. Taga T. Watanabe Y. Matsuda T. (1986). Complementary DNA for a Novel Human Interleukin (BSF-2) that Induces B Lymphocytes to Produce Immunoglobulin. Nature 324, 73–76. 10.1038/324073a0 3491322

[B16] Ho M. S. (2019). Microglia in Parkinson's Disease. Adv. Exp. Med. Biol. 1175, 335–353. 10.1007/978-981-13-9913-8_13 31583594

[B17] Huang C. Ma R. Sun S. Wei G. Fang Y. Liu R. (2008). JAK2-STAT3 Signaling Pathway Mediates Thrombin-Induced Proinflammatory Actions of Microglia *In Vitro* . J. Neuroimmunol 204, 118–125. 10.1016/j.jneuroim.2008.07.004 18710787

[B18] Jenab S. Quinones-Jenab V. (2002). The Effects of Interleukin-6, Leukemia Inhibitory Factor and Interferon-Gamma on STAT DNA Binding and C-Fos mRNA Levels in Cortical Astrocytes and C6 Glioma Cells. Neuro Endocrinol. Lett. 23, 325–328. 12195235

[B19] Li L. Lu J. Tay S. S. Moochhala S. M. He B. P. (2007). The Function of Microglia, Either Neuroprotection or Neurotoxicity, Is Determined by the Equilibrium Among Factors Released from Activated Microglia *In Vitro* . Brain Res. 1159, 8–17. 10.1016/j.brainres.2007.04.066 17572395

[B20] Li L. Wan G. Han B. Zhang Z. (2018a). Echinacoside Alleviated LPS-Induced Cell Apoptosis and Inflammation in Rat Intestine Epithelial Cells by Inhibiting the mTOR/STAT3 Pathway. Biomed. Pharmacother. 104, 622–628. 10.1016/j.biopha.2018.05.072 29803175

[B21] Li L. Wang Y. Qin X. Zhang J. Zhang Z. (2018b). Echinacoside Protects Retinal Ganglion Cells from Ischemia/reperfusion-Induced Injury in the Rat Retina. Mol. Vis. 24, 746–758. 30581281 PMC6279312

[B22] Li W. Y. Li F. M. Zhou Y. F. Wen Z. M. Ma J. Ya K. (2016). Aspirin Down Regulates Hepcidin by Inhibiting NF-Κb and IL6/JAK2/STAT3 Pathways in BV-2 Microglial Cells Treated with Lipopolysaccharide. Int. J. Mol. Sci. 17 (12), 1921. 10.3390/ijms17121921 PMC5187761 27999284

[B23] Li X. Z. Bai L. M. Yang Y. P. Luo W. F. Hu W. D. Chen J. P. (2009). Effects of IL-6 Secreted from Astrocytes on the Survival of Dopaminergic Neurons in Lipopolysaccharide-Induced Inflammation. Neurosci. Res. 65, 252–258. 10.1016/j.neures.2009.07.007 19647022

[B24] Ma J. Gao J. Niu M. Zhang X. Wang J. Xie A. (2020). P2X4Roverexpressionupregulates Interleukin -6andexacerbates 6-OHDA-Induceddopaminergic Degeneration inaRat Model ofPD. Front. inAging Neurosci. 12, 580068. 10.3389/fnagi.2020.580068 PMC7671967 33328961

[B25] Mandal T. Bhowmik A. Chatterjee A. Chatterjee U. Chatterjee S. Ghosh M. K. (2014). Reduced Phosphorylation of Stat3 at Ser-727 Mediated by Casein Kinase 2 - Protein Phosphatase 2A Enhances Stat3 Tyr-705 Induced Tumorigenic Potential of Glioma Cells. Cell Signal 26 (8), 1725–1734. 10.1016/j.cellsig.2014.04.003 24726840

[B26] Mertens C. Darnell J. E. (2007). SnapShot: JAK-STAT Signaling. Cell 131, 612. 10.1016/j.cell.2007.10.033 17981126

[B27] Ng Y. P. Cheung Z. H. Ip N. Y. (2006). STAT3 as a Downstream Mediator of Trk Signaling and Functions. J. Biol. Chem. 281, 15636–15644. 10.1074/jbc.M601863200 16611639

[B28] Pang Y. Fan L. W. Zheng B. Cai Z. Rhodes P. G. (2006). Role of Interleukin-6 in Lipopolysaccharide-Induced Brain Injury and Behavioral Dysfunction in Neonatal Rats. Neuroscience 141, 745–755. 10.1016/j.neuroscience.2006.04.007 16713113

[B29] Paris A. J. Hayer K. E. Oved J. H. Avgousti D. C. Toulmin S. A. Zepp J. A. (2020). STAT3-BDNF-TrkB Signalling Promotes Alveolar Epithelial Regeneration after Lung Injury. Nat. Cel Biol 22 (10), 1197–1210. 10.1038/s41556-020-0569-x PMC8167437 32989251

[B30] Pellegrino M. J. Habecker B. A. (2013). STAT3 Integrates Cytokine and Neurotrophin Signals to Promote Sympathetic Axon Regeneration. Mol. Cel Neurosci 56, 272–282. 10.1016/j.mcn.2013.06.005 PMC3791163 23831387

[B31] Planas A. M. Gorina R. Chamorro A. (2006). Signalling Pathways Mediating Inflammatory Responses in Brain Ischaemia. Biochem. Soc. Trans. 34, 1267–1270. 10.1042/BST0341267 17073799

[B32] Poewe W. Seppi K. Tanner C. M. Halliday G. M. Brundin P. Volkmann J. (2017). Parkinson Disease. Nat. Rev. Dis. Primers 3, 17013. 10.1038/nrdp.2017.13 28332488

[B33] Postuma R. B. Berg D. Stern M. Poewe W. Olanow C. W. Oertel W. (2015). MDS Clinical Diagnostic Criteria for Parkinson's Disease. Mov Disord. 30 (12), 1591–1601. 10.1002/mds.26424 26474316

[B34] Qin H. Buckley J. A. Li X. Liu Y. Fox T. H. Meares G. P. (2016). Inhibition of the JAK/STAT Pathway Protects against α-Synuclein-Induced Neuroinflammation and Dopaminergic Neurodegeneration. J. Neurosci. 36, 5144–5159. 10.1523/JNEUROSCI.4658-15.2016 27147665 PMC6123006

[B36] Qiu Z. Sweeney D. D. Netzeband J. G. Gruol D. L. (1998). Chronic Interleukin-6 Alters NMDA Receptor-Mediated Membrane Responses and Enhances Neurotoxicity in Developing CNS Neurons. J. Neurosci. 18, 10445–10456. 10.1523/jneurosci.18-24-10445.1998 9852582 PMC6793367

[B37] Rabiei Z. Solati K. Amini-Khoei H. (2019). Phytotherapy in Treatment of Parkinson's Disease: a Review. Pharm. Biol. 57 (1), 355–362. 10.1080/13880209.2019.1618344 31141426 PMC6542178

[B38] Rojo A. I. Innamorato N. G. Martín-Moreno A. M. De Ceballos M. L. Yamamoto M. Cuadrado A. (2010). Nrf2 Regulates Microglial Dynamics and Neuroinflammation in Experimental Parkinson's Disease. Glia 58 (5), 588–598. 10.1002/glia.20947 19908287

[B39] Satoh T. Nakamura S. Taga T. Matsuda T. Hirano T. Kishimoto T. (1988). Induction of Neuronal Differentiation in PC12 Cells by B-Cell Stimulatory Factor 2/interleukin 6. Mol. Cel Biol 8, 3546–3549. 10.1128/mcb.8.8.3546 PMC363593 3264880

[B40] Satriotomo I. Bowen K. K. Vemuganti R. (2006). JAK2 and STAT3 Activation Contributes to Neuronal Damage Following Transient Focal Cerebral Ischemia. J. Neurochem. 98 (5), 1353–1368. 10.1111/j.1471-4159.2006.04051.x 16923154

[B41] Shi X. Zhang H. Paddon H. Lee G. Cao X. Pelech S. (2006). Phosphorylation of STAT3 Serine-727 by Cyclin-dependent Kinase 1 Is Critical for Nocodazole-Induced Mitotic Arrest. Biochemistry 45 (18), 5857–5867. 10.1021/bi052490j 16669628

[B42] Sliter D. A. Martinez J. Hao L. Chen X. Sun N. Fischer T. D. (2018). Parkin and PINK1 Mitigate STING-Induced Inflammation. Nature 561 (7722), 258–262. 10.1038/s41586-018-0448-9 30135585 PMC7362342

[B43] Spooren A. Kolmus K. Laureys G. Clinckers R. De Keyser J. Haegeman G. (2011). Interleukin-6, a Mental Cytokine. Brain Res. Rev. 67, 157–183. 10.1016/j.brainresrev.2011.01.002 21238488

[B44] Sriram K. Benkovic S. A. Hebert M. A. Miller D. B. O'Callaghan J. P. (2004). Induction of Gp130-Related Cytokines and Activation of JAK2/STAT3 Pathway in Astrocytes Precedes Up-Regulation of Glial Fibrillary Acidic Protein in the 1-Methyl-4-Phenyl-1,2,3,6-Tetrahydropyridine Model of Neurodegeneration: Key Signaling Pathway for Astrogliosis *In Vivo* . J. Biol. Chem. 279 (19), 19936–19947. 10.1074/jbc.M309304200 14996842

[B45] Tanaka T. Narazaki M. Kishimoto T. (2014). IL-6 in Inflammation, Immunity, and Disease. Cold Spring Harb Perspect. Biol. 6 (10), a016295. 10.1101/cshperspect.a016295 25190079 PMC4176007

[B46] Wang A. L. Liou Y. M. Pawlak C. R. Ho Y. J. (2010). Involvement of NMDA Receptors in Both MPTP-Induced Neuroinflammation and Deficits in Episodic-like Memory in Wistar Rats. Behav. Brain Res. 208 (1), 38–46. 10.1016/j.bbr.2009.11.006 19900486

[B47] Wang B. Wang X.-q. Yang S.-s. Liu X. Feng D.-y. Lu F.-f. Zhu Y.-q. Lu D. Tao L. Ge S.-n. Gao L. Qu Y. Gao G.-d. (2016). Neuroprotective Effects of Nitidine in Parkinson's Disease Models through Inhibiting Microglia Activation: Role of the Jak2-Stat3 Pathway. RSC Adv. 6, 71328–71337. 10.1039/c6ra11759g

[B48] Wang Q. Liu Y. Zhou J. (2015). Neuroinflammation in Parkinson's Disease and its Potential as Therapeutic Target. Transl Neurodegener 4, 19. 10.1186/s40035-015-0042-0 26464797 PMC4603346

[B49] Wang X. Liu Z. Wang F. (2021). MicroRNA-93 Blocks Signal Transducers and Activator of Transcription 3 to Reduce Neuronal Damage in Parkinson's Disease. Neurochem. Res. 46 (7), 1859–1868. 10.1007/s11064-021-03333-x 33900518

[B50] Wang Y. H. Xuan Z. H. Tian S. Du G. H. (20152015). Echinacoside Protects against 6-Hydroxydopamine-Induced Mitochondrial Dysfunction and Inflammatory Responses in PC12 Cells via Reducing ROS Production. Evid. Based Complement. Alternat Med. 2015, 189239. 10.1155/2015/189239 PMC4348598 25788961

[B51] Yao S. Li L. Sun X. Hua J. Zhang K. Hao L. (2019). FTY720 Inhibits MPP+-induced Microglial Activation by Affecting NLRP3 Inflammasome Activation. J. Neuroimmune Pharmacol. 14 (3), 478–492. 10.1007/s11481-019-09843-4 31069623

[B52] Yin L. Dai Q. Jiang P. Zhu L. Dai H. Yao Z. Liu H. Ma X. Qu L. Jiang J. (2018). Manganese Exposure Facilitates Microglial JAK2-STAT3 Signaling and Consequent Secretion of TNF-A and IL-1β to Promote Neuronal Death. Neurotoxicology 64, 195–203. 10.1016/j.neuro.2017.04.001 28385490

[B53] Zhang J. Zhang Z. Xiang J. Cai M. Yu Z. Li X. (2017). Neuroprotective Effects of Echinacoside on Regulating the Stress-Active p38MAPK and NF-Κb P52 Signals in the Mice Model of Parkinson's Disease. Neurochem. Res. 42, 975–985. 10.1007/s11064-016-2130-7 27981472

[B54] Zhang Z. N. Hui Z. Chen C. Liang Y. Tang L. L. Wang S. L. (2021). Neuroprotective Effects and Related Mechanisms of Echinacoside in MPTP-Induced PD Mice. Neuropsychiatr. Dis. Treat. 17, 1779–1792. 10.2147/NDT.S299685 34113108 PMC8184243

[B55] Zhou L. Too H. P. (2011). Mitochondrial Localized STAT3 Is Involved in NGF Induced Neurite Outgrowth. PLoS One 6, e21680. 10.1371/journal.pone.0021680 21738764 PMC3124549

[B56] Zhou L. Yao M. Tian Z. Song Y. Sun Y. Ye J. (2020). Echinacoside Attenuates Inflammatory Response in a Rat Model of Cervical Spondylotic Myelopathy via Inhibition of Excessive Mitochondrial Fission. Free Radic. Biol. Med. 152, 697–714. 10.1016/j.freeradbiomed.2020.01.014 32014501

[B57] Zhu H. Jian Z. Zhong Y. Ye Y. Zhang Y. Hu X. (2021). Janus Kinase Inhibition Ameliorates Ischemic Stroke Injury and Neuroinflammation through Reducing NLRP3 Inflammasome Activation via JAK2/STAT3 Pathway Inhibition. Front. Immunol. 12, 714943. 10.3389/fimmu.2021.714943 34367186 PMC8339584

[B58] Zhu M. Lu C. Li W. (2013). Transient Exposure to Echinacoside Is Sufficient to Activate Trk Signaling and Protect Neuronal Cells from Rotenone. J. Neurochem. 124 (4), 571–580. 10.1111/jnc.12103 23189969

