# Supplementary figures and images for: Echinacoside Protects Dopaminergic Neurons Through Regulating IL-6/JAK2/STAT3 Pathway in Parkinson’s Disease Model
Source: Front Pharmacol. 2022 Feb 25;13:848813. doi: 10.3389/fphar.2022.848813 (PMC8914071; doi:10.3389/fphar.2022.848813)

# Graphical Abstract

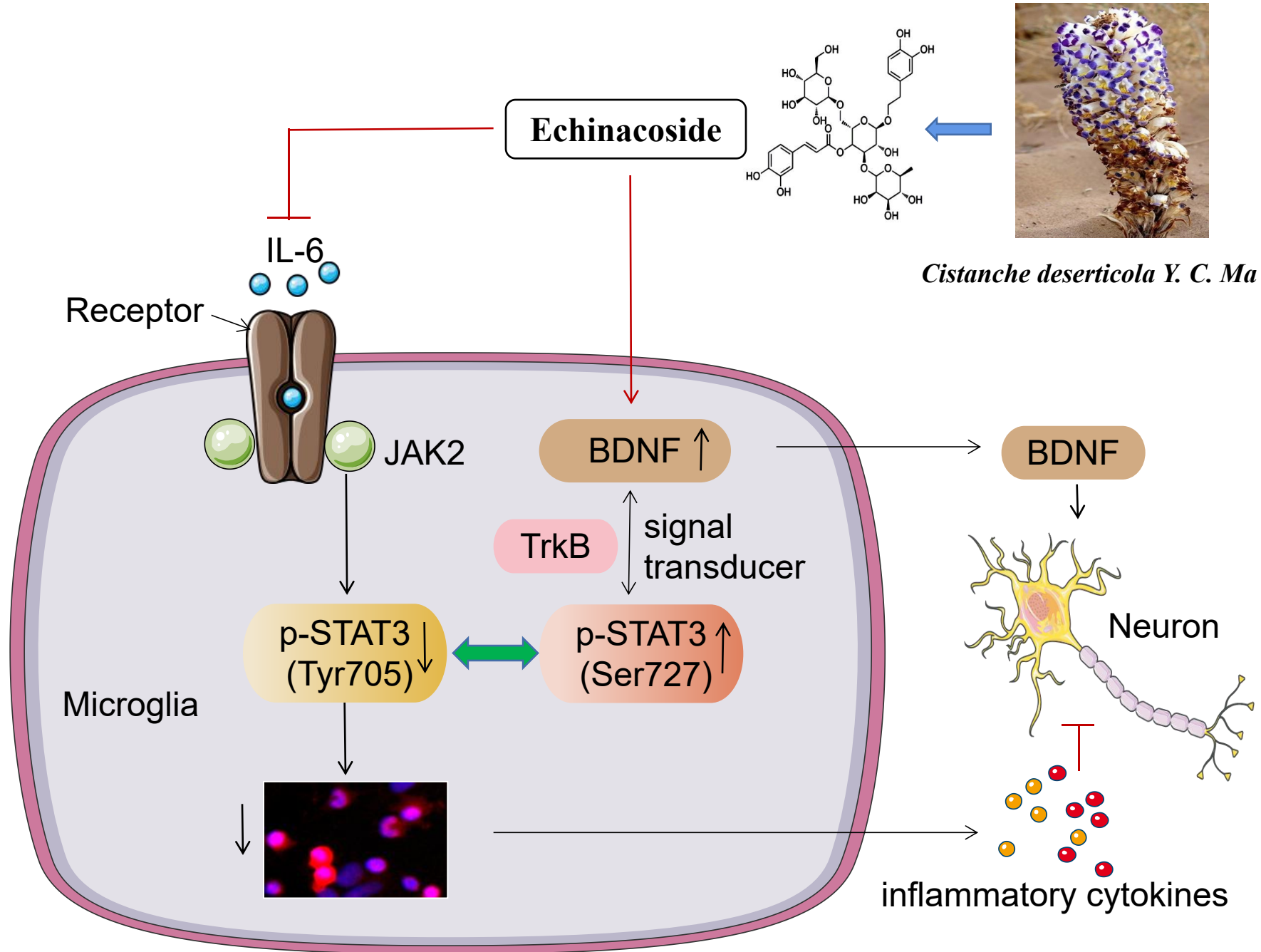

Supplement: Supplementary file 1 [file DataSheet1.pdf]

Supplement:

Western blot with protein marker

Figure 2A

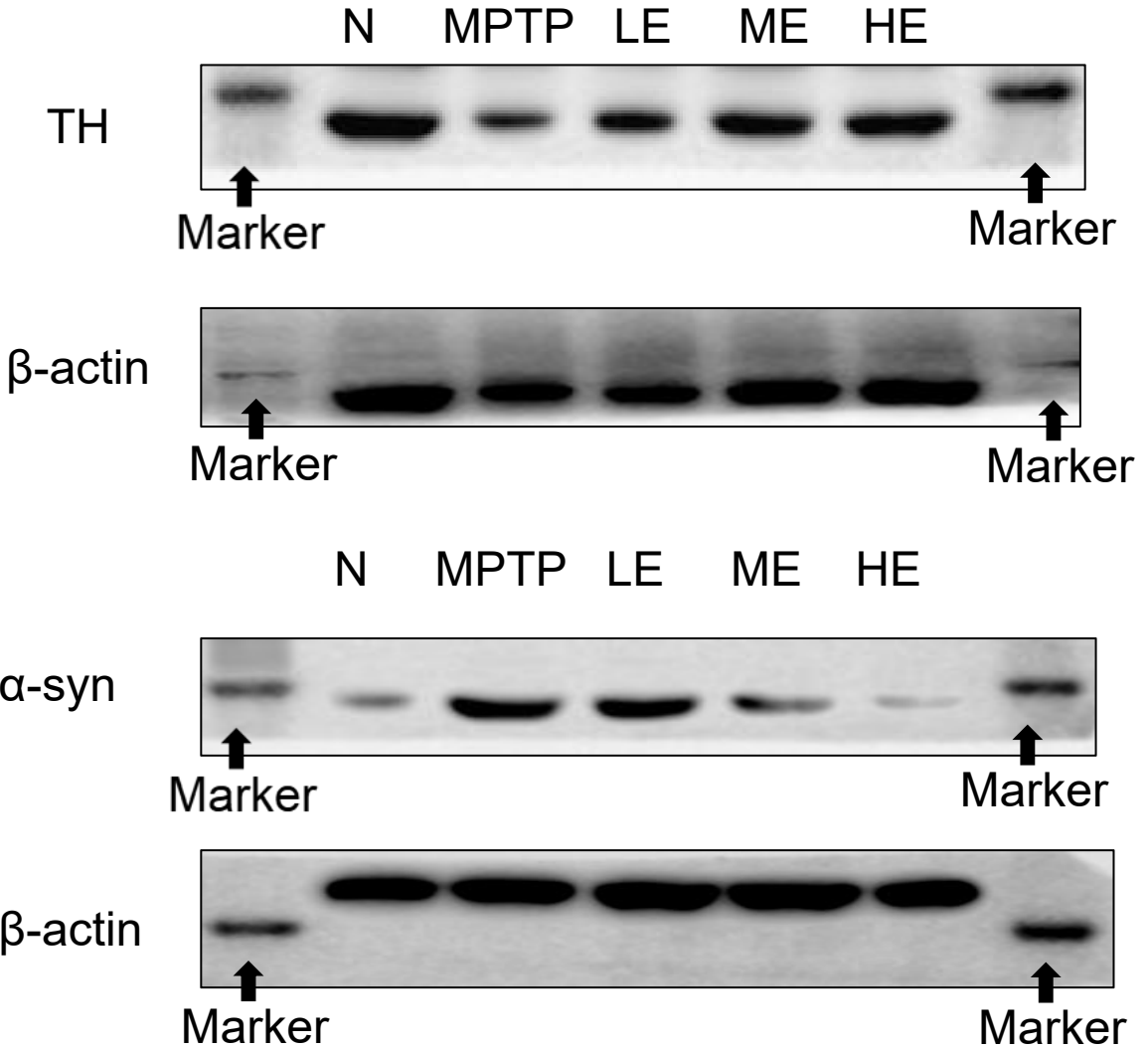

Figure 4A

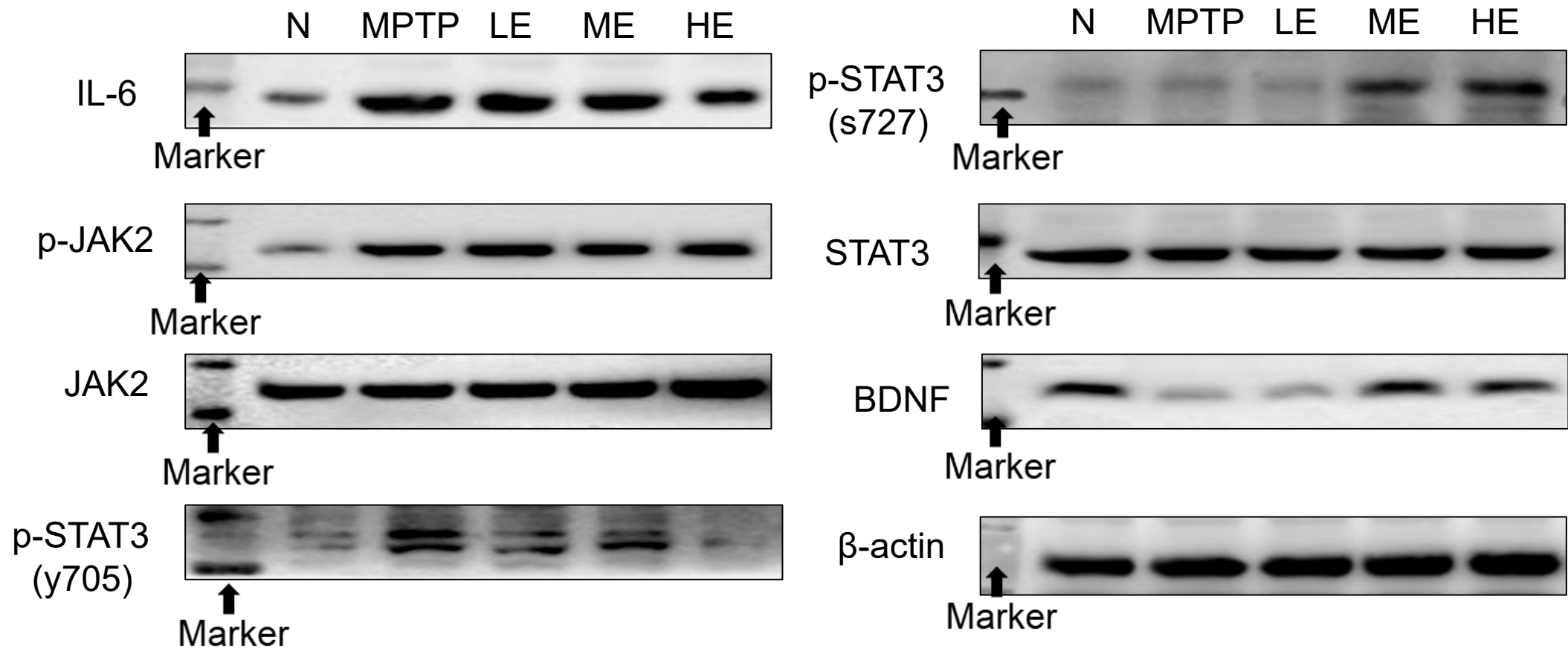

Figure 6A

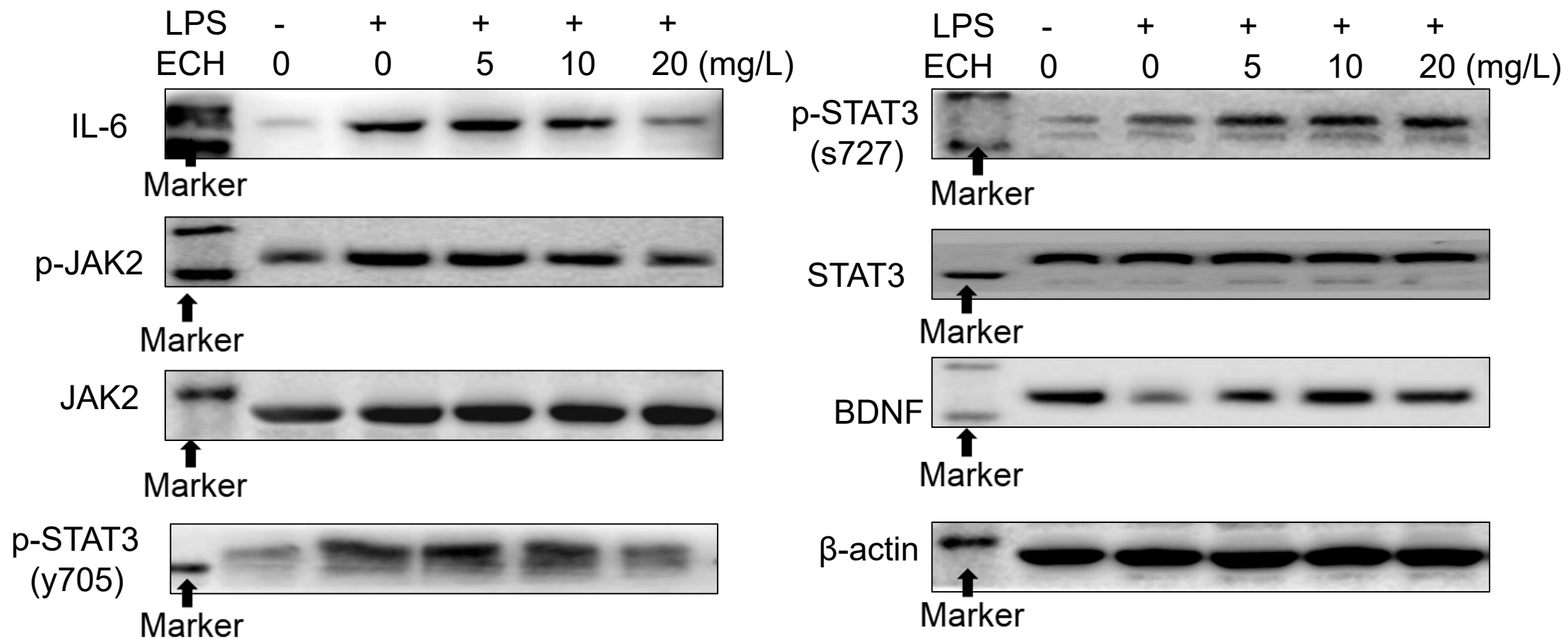

Figure 6H

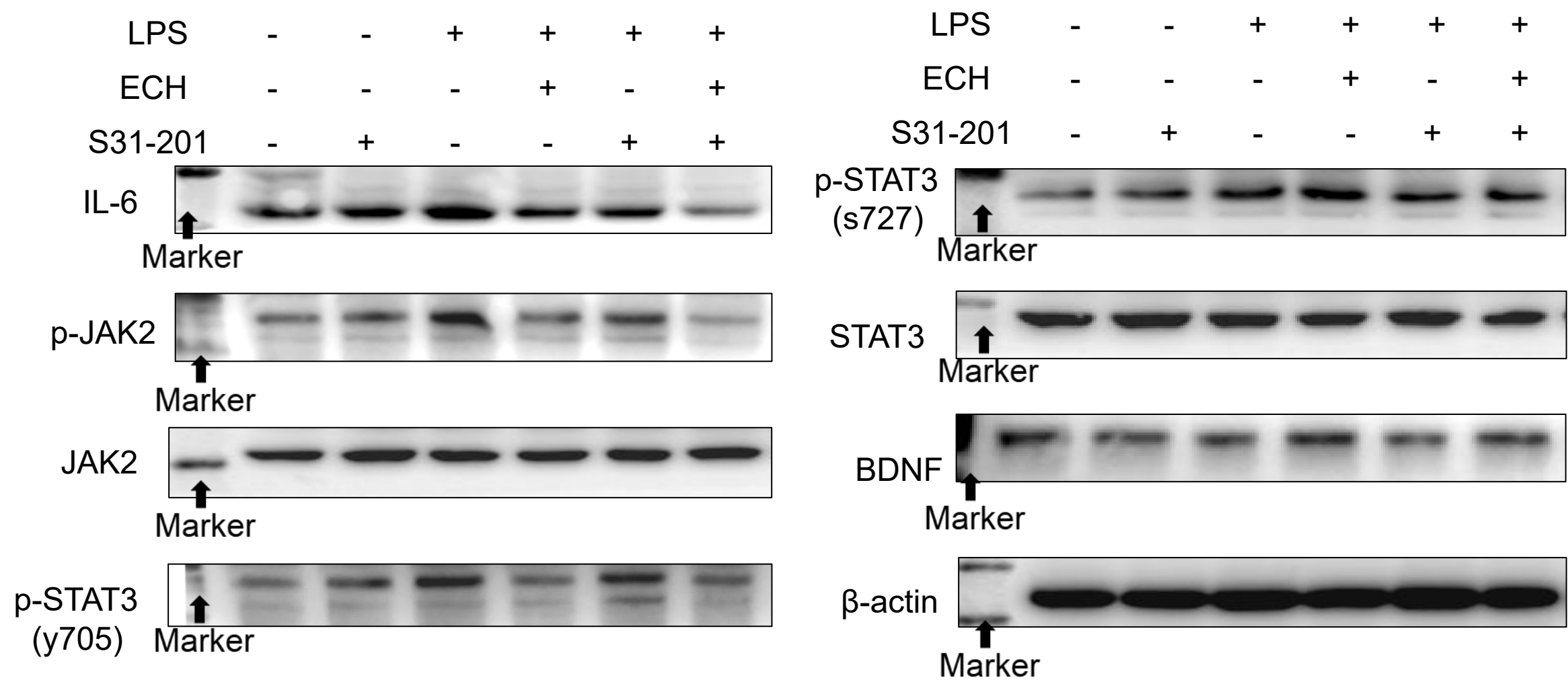

Supplement: Supplementary file 2 [file Image1.pdf]
